# Clinical delineation, sex differences, and genotype–phenotype correlation in pathogenic *KDM6A* variants causing X-linked Kabuki syndrome type 2

**DOI:** 10.1038/s41436-021-01119-8

**Published:** 2021-03-05

**Authors:** Víctor Faundes, Stephanie Goh, Rhoda Akilapa, Heidre Bezuidenhout, Hans T. Bjornsson, Lisa Bradley, Angela F. Brady, Elise Brischoux-Boucher, Han Brunner, Saskia Bulk, Natalie Canham, Declan Cody, Maria Lisa Dentici, Maria Cristina Digilio, Frances Elmslie, Andrew E. Fry, Harinder Gill, Jane Hurst, Diana Johnson, Sophie Julia, Katherine Lachlan, Robert Roger Lebel, Melissa Byler, Eric Gershon, Edmond Lemire, Maria Gnazzo, Francesca Romana Lepri, Antonia Marchese, Meriel McEntagart, Julie McGaughran, Seiji Mizuno, Nobuhiko Okamoto, Claudine Rieubland, Jonathan Rodgers, Erina Sasaki, Emmanuel Scalais, Ingrid Scurr, Mohnish Suri, Ineke van der Burgt, Naomichi Matsumoto, Noriko Miyake, Valérie Benoit, Damien Lederer, Siddharth Banka

**Affiliations:** 1 Division of Evolution & Genomic Sciences, School of Biological Sciences, Faculty of Biology, Medicine and Health, University of Manchester, Manchester, UK; 2 Laboratorio de Genética y Enfermedades Metabólicas, Instituto de Nutrición y Tecnología de los Alimentos (INTA), Universidad de Chile, Santiago, Chile; 3 School of Medical Sciences, Faculty of Biology, Medicine and Health, University of Manchester, Manchester, UK; 4 NW Thames Regional Genetics Service, Northwick Park Hospital, Harrow, UK; 5 Clinical Unit of Medical Genetics and Genetic Counselling, Tygerberg Academic Hospital, Cape Town, South Africa; 6 Division of Molecular Biology and Human Genetics, Faculty of Medicine and Health Sciences, Stellenbosch University, Cape Town, South Africa; 7 McKusick-Nathans Department of Genetic Medicine, Johns Hopkins University School of Medicine, Baltimore, MD USA; 8 Faculty of Medicine, University of Iceland, Reykjavik, Iceland; 9 Department of Clinical Genetics, Children’s Health Ireland at Crumlin, Dublin, Ireland; 10 Centre de Génétique Humaine, Centre Hospitalier et Universitaire, Université de Franche-Comté, Besançon, France; 11 Department of Human Genetics, Radboud University Medical Center, Nijmegen, The Netherlands; 12 Centre de Génétique Humaine, CHU de Liège, Liège, Belgium; 13 Liverpool Centre for Genomic Medicine, Liverpool Women’s Hospital, Crown Street, Liverpool, UK; 14 Medical Genetics Unit, Academic Department of Pediatrics, Bambino Gesù Children’s Hospital, IRCCS, Rome, Italy; 15 SW Thames Regional Genetics Service, St George’s, University of London, London, UK; 16 Institute of Medical Genetics, University Hospital of Wales, Heath Park, Cardiff, UK; 17 NE Thames Genetics Service, Great Ormond Street Hospital, London, UK; 18 Sheffield Clinical Genetics Service, Sheffield Children’s NHS Foundation Trust, Northern General Hospital, Sheffield, UK; 19 Departments of Pathology, Neurosurgery, Oncopediatry, Genetics and Molecular Biology, Toulouse University Hospital, Toulouse, France; 20 Wessex Clinical Genetics Service and Division of Human Genetics, Princess Anne Hospital, Southampton, UK; 21 Department of Pediatrics, Section of Medical Genetics, SUNY Upstate Medical University, Syracuse, NY USA; 22 Department of Pediatrics, Yale New Haven Health, New Haven, CT USA; 23 Department of Pediatrics, Royal University Hospital, University of Saskatchewan, Saskatoon, SK Canada; 24 Laboratory of Medical Genetics, Bambino Gesù Children’s Hospital, IRCCS, Rome, Italy; 25 Service de Pédiatrie, Centre Hospitalier Régional de Namur, Namur, Belgium; 26 Genetic Health Queensland c/-Royal Brisbane and Women’s Hospital, Herston, QLD Australia; 27 Department of Clinical Genetics, Central Hospital, Aichi Developmental Disability Center, Aichi, Japan; 28 Department of Medical Genetics, Osaka Women’s and Children’s Hospital, Osaka, Japan; 29 Department of Molecular Medicine, Osaka Women’s and Children’s Hospital, Osaka, Japan; 30 Division of Human Genetics, Department of Pediatrics, Inselspital, Bern University Hospital, University of Bern, Bern, Switzerland; 31 Department of Pediatric Neurology, National Hospital, Luxembourg City, Luxembourg; 32 Clinical Genetics, University Hospitals Bristol, Bristol, UK; 33 Nottingham Clinical Genetics Service, City Hospital Campus, Nottingham, UK; 34 Department of Human Genetics, Radboud University Medical Center, Donders Institute for Brain, Cognition and Behaviour, Nijmegen, The Netherlands; 35 Department of Human Genetics, Yokohama City University Graduate School of Medicine, Yokohama, Japan; 36 Centre de Génétique Humaine, Institut de Pathologie et de Génétique, Gosselies, Belgium; 37 Manchester Centre for Genomic Medicine, St Mary’s Hospital, Manchester University NHS Foundation Trust, Health Innovation Manchester, Manchester, UK

## Abstract

**Purpose:**

The variant spectrum and the phenotype of X-linked Kabuki syndrome type 2 (KS2) are poorly understood.

**Methods:**

Genetic and clinical details of new and published individuals with pathogenic *KDM6A* variants were compiled and analyzed.

**Results:**

Sixty-one distinct pathogenic *KDM6A* variants (50 truncating, 11 missense) from 80 patients (34 males, 46 females) were identified. Missense variants clustered in the TRP 2, 3, 7 and Jmj-C domains. Truncating variants were significantly more likely to be de novo. Thirteen individuals had maternally inherited variants and one had a paternally inherited variant. Neonatal feeding difficulties, hypoglycemia, postnatal growth retardation, poor weight gain, motor delay, intellectual disability (ID), microcephaly, congenital heart anomalies, palate defects, renal malformations, strabismus, hearing loss, recurrent infections, hyperinsulinism, seizures, joint hypermobility, and gastroesophageal reflux were frequent clinical findings. Facial features of over a third of patients were not typical for KS. Males were significantly more likely to be born prematurely, have shorter stature, and severe developmental delay/ID.

**Conclusion:**

We expand the *KDM6A* variant spectrum and delineate the KS2 phenotype. We demonstrate that the variability of the KS2 phenotypic depends on sex and the variant type. We also highlight the overlaps and differences between the phenotypes of KS2 and KS1.

## INTRODUCTION

Kabuki syndrome (KS, MIM 147920 and MIM 300867) is one of the commonest congenital disorders caused by variants in genes encoding histone lysine methylases and demethylases.^1^ It is characterized by a distinctive facies (long palpebral fissures with eversion of the lateral third of the lower eyelid; arched and broad eyebrows with the lateral third displaying notching or sparseness; large, prominent, or cupped ears; and short columella with depressed nasal tip), developmental delay and/or intellectual disability (ID), and several structural (e.g., congenital heart defects, genitourinary malformations) and functional anomalies (e.g., increased susceptibility to infections, endocrine disorders, deafness).^2^ The majority of patients with KS have loss-of-function (LoF), mostly de novo, variants in *KMT2D* (formerly known as *MLL2* and *ALR*) (KS1, MIM 147920).^3,4^ *KMT2D* is located on chromosome 12, and encodes lysine (K)-specific methyltransferase 2D, which catalyzes the trimethylation of the lysine 4 on histone 3 (H3K4), promoting the expression of its target genes.^5^

In two girls and a boy with KS-like features, Lederer et al.^6^ identified de novo X-chromosome deletions that encompassed *KDM6A* (formerly known as *UTX*) (KS2, MIM 300867). Miyake et al.^7^ and Banka et al.^8^ subsequently identified pathogenic point variants in *KDM6A* by targeted sequencing in cohorts of patients clinically suspected to have KS and showed that *KDM6A* variants account for ~5% cases of KS. *KDM6A* partially escapes X-inactivation.^6,9^ The canonical transcript (GenBank NM_021140.3; Ensembl ENST00000377967.8) has 29 exons and encodes a H3K27 demethylase of 1,401 amino acids (UniProtKB entry O15550). The Jumonji-C (Jmj-C) domain is the catalytic lysine demethylase domain, which is situated toward the C-terminus of the protein.^10^ Toward its N-terminus, the protein includes eight tetratricopeptide repeats (TPR) that may contribute indirectly to substrate binding, but their precise function is unknown.

Most published patients with *KDM6A* variants have been primarily ascertained based on phenotype as part of short case series or single case reports.^7,8,11–16^ It is known that the phenotype of patients with KS2 can be atypical for KS.^8,17^ Application of next-generation sequencing (NGS)–based diagnostics is enabling identification of patients with *KDM6A* variants and atypical phenotypes.^17^ Accurate clinical correlation of *KDM6A* variants in absence of typical phenotype can be challenging (especially for inherited missense variants) because the *KDM6A* variant and clinical spectra are not well defined. Also, lack of genotype–phenotype correlation studies makes estimation of prognosis challenging. Here we present a large case series of patients with *KDM6A* variants that helps broaden the variant spectrum, delineates the range of the associated clinical features, enables comparison of phenotypes of affected males and females, and allows genotype–phenotype correlations.

## MATERIALS AND METHODS

### Ascertainment

#### New patients

Procedures were in accordance with local ethical standards. Individuals with rare *KDM6A* variants were mainly identified via the Manchester Centre for Genomic Medicine, UK (Research Ethics Committee Approval [RECA] 02/CM/238), Centre de Génétique Humaine, Institut de Pathologie et de Génétique, Belgium (RECA 050612), Epigenetic and Chromatin Clinic at Johns Hopkins Hospital, USA (IRB approval: NA_00079185), and the Deciphering Developmental Disorders (DDD) study (RECAs 10/H0305/83 and GEN/284/12).^18^

#### Previously reported individuals

All articles indexed in PubMed between 1 January 2012 (year in which in *KDM6A* variants were first associated with KS^6^) and 31 March 2019 were retrieved using the following terms “*KDM6A* NOT(cancer)” OR “Kabuki NOT(Kabuki[Author])” OR “Kabuki make-up,” OR “Niikawa-Kuroki.” Articles on KS1 or papers that did not provide the phenotype of affected individuals were excluded following title and abstract review. Full texts of the remaining articles were reviewed. The criteria for including published patients in the present study were (1) availability of clinical details, (2) unambiguous description of the *KDM6A* variant, and (3) not duplicated from any previous report. Exclusion criteria were (1) patients with copy-number losses that encompassed additional known developmental disorder related genes, and (2) patients with additional known genetic developmental disorders.

#### Variant analysis

All detected point variants were harmonized according to the canonical *KDM6A* transcript NM_021140.3 using MutationTaster2 (http://www.mutationtaster.org/). InterVar (http://wintervar.wglab.org/) was used to apply the 2015 American College of Medical Genetics and Genomics/Association for Molecular Pathology (ACMG/AMP) guidelines for variant interpretation.^19^ Protein altering variants (PAVs) were analyzed by the Variant Effect Predictor (http://grch37.ensembl.org/Homo_sapiens/Tools/VEP) to obtain minor allele frequencies (MAFs) in controls, exon location, in silico predictions, previous reports, and evolutionary conservation. Alamut® Visual 2.11 (Interactive Biosoftware, France) and UniProtKB (https://www.uniprot.org/uniprot/O15550) were used for exon-skipping analyses and determining affected domains, respectively. PAVs with decreased in vitro demethylation were obtained from the work of Shpargel et al.^20^ To analyze copy-number variants (CNVs) encompassing *KDM6A*, the University of California–Santa Cruz (UCSC) Genome Browser (GRCh37) was used.

### Data collection

Clinical data were collected on individuals with pathogenic or likely pathogenic (just pathogenic hereafter) *KDM6A* variants. The clinical proforma was completed by recruiting clinicians for new patients. V.F. (the first author of the present study) completed the proforma for previously published patients.

### Statistical analyses

For calculation of frequencies of individual features, we excluded individuals for whom that feature was coded as “UNKNOWN” (which includes instances where presence or absence of a particular feature was not clearly documented or where a feature may not be applicable due to the individual’s sex or age) in the clinical proforma (Supplementary Table S1). Absolute and relative frequencies (expressed as *n*[%]) were used for describing categorical variables, whereas medians (m), interquartile ranges (IQR) and minimum (min) and maximum (max) were used for describing continuous variables. Chi-square/Fisher’s exact and Wilcoxon–Mann–Whitney test was applied to study categorical and continuous variables respectively. Two-tailed/adjusted *p* value <0.05 was considered as significant for all statistical analyses, which were carried out using the IBM© SPSS© Version 25 software.

## RESULTS

### Ascertainment

We identified 36 new patients (18 males/18 females) with hetero/hemizygous *KDM6A* variants (Supplementary Table S1). We also identified 49 patients (19 males and 30 females) with *KDM6A* variants from 17 peer-reviewed articles (Supplementary Figure S1 and Supplementary Table S1).

### Variants

**Fig. 1:**
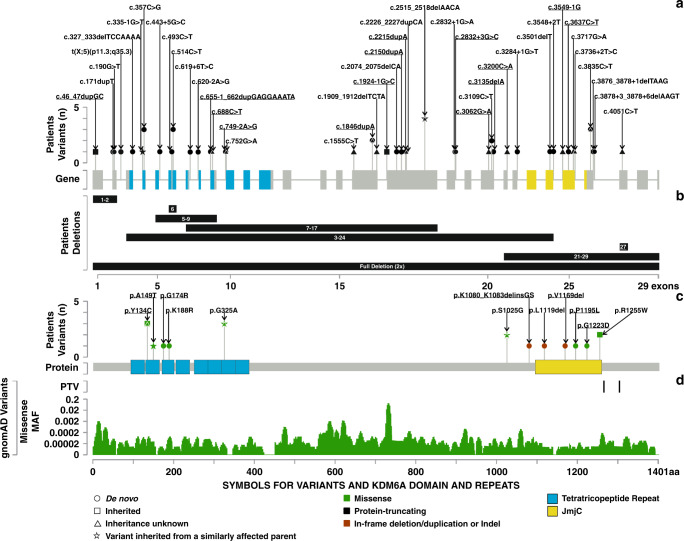
Spectrum of pathogenic *KDM6A* variants. (**a**) Protein-truncating variants (PTVs) described in this study. The *x*-axis shows the exon–intron locations and the *y*-axis shows the number of variants detected. Novel PTVs are underlined. (**b**) Deletions described in this study. The *x*-axis shows the exons affected. Novel deletions are underlined. (**c**) Protein-altering variants (PAVs) described in this study. The *x*-axis shows the protein location and the *y*-axis shows the number of variants detected. Novel PAVs are underlined. (**d**) Control variants seen in gnomAD, PTVs at the top and missense variants at the bottom. The *x*-axis shows the protein location and *y*-axis shows the minor allele frequencies of missense variants. MAF minor allele frequency.

In total, we compiled 66 distinct *KDM6A* variants from these 85 patients (37 males, 48 females) from 78 families. This included 50 PTVs in 62 individuals (26 males, 36 females) from 59 families (Fig. 1a, b) and 16 PAVs in 23 individuals (11 males, 12 females) (Fig. 1c) from 19 families.

All the PTVs were classified as pathogenic. We identified 15 novel PTVs. Fifteen of the 50 PTVs (30%) were nonsense, 14 (28%) affected canonical splice sites, 12 (24%) were frameshift, 8 (16%) were gross deletions, and 1 (2%) resulted from a gene-disrupting chromosome translocation (Fig. 1a, b, and Supplementary Figure S2A). In 42 patients (13 males, 29 females) the *KDM6A* PTVs had occurred de novo, whereas 6 patients (5 males, 1 female) had inherited their variants from mothers. The inheritance of 14 PTVs (8 males, 6 females) was unknown (Supplementary Figure S2B).

Of 16 PAVs, 12 were classed as pathogenic or likely pathogenic, 3 were classed as variants of uncertain significance (VUS), and 1 was classed as likely benign (Supplementary Table S2 and Supplementary Figure S2D). Patients with VUS or likely benign variants (five patients in total) were excluded from subsequent analyses. Thirteen of the 16 PAVs (81.3%) were missense, 2 (12.5%) were in-frame deletions, and 1 (6.2%) was an indel (Fig. 1c and Supplementary Figure S2A). Eight PAVs (2 males, 6 females) had occurred de novo. Ten patients (7 males, 3 females) had inherited their PAVs from their mothers (in one case mother is mosaic for p.Arg1255Trp) and one female had inherited her p.Ala149Thr *KDM6A* PAV from her father. Inheritance of 4 PAVs (2 males, 2 females) was unknown (Supplementary Figure S2B).

### Clinical delineation of KS2

In total, we analyzed clinical findings from 80 individuals with pathogenic or likely pathogenic *KDM6A* variants (Table 1). In this cohort 57.5% (*n* = 46) were females, and the median age at last examination was 7 years (min = 0.21, max = 37). The youngest male patient was 2.5 months old and the oldest male patient was 37 years old. The youngest female patient was 3.5 months old and the oldest female patient was 37 years old.

**Table 1:** Clinical findings and their frequencies or distributions across 80 new and published cases of Kabuki syndrome type 2.

Clinical finding^a^	Frequency or distribution
Sex (males/females, *n*[%]) (*n* = 80)	34 (42.5)/46 (57.5)
Age at last examination (years, m[IQR]) (*n* = 57)	7 (3.25; 13)
Typical craniofacial dysmorphisms^b^ (*n*[%]) (*n* = 80)	51 (63.7)
Growth parameters^c^	
Weight SD (m[IQR]) (*n* = 43)	−1.43 (−2.65; 0)
Height SD (m[IQR]) (*n* = 46)	−2 (−2.82; −1.16)
HC SD (m[IQR]) (*n* = 43)	−2.34 (−3; −0.61)
Antenatal and neonatal features	
Abnormal pregnancy findings (*n*[%]) (*n* = 27)	10 (37)
Gestation duration (weeks, m[IQR]) (*n* = 45)	38 (36; 39)
Birth weight SD (m[IQR]) (*n* = 44)	0.01 (−1.19; 0.69)
Birth length SD (m[IQR]) (*n* = 18)	−0.64 (−1.31; 0.83)
Birth HC SD (m[IQR]) (*n* = 20)	−0.15 (−1.25; 0.31)
Neonatal hypotonia (*n*[%]) (*n* = 44)	35 (79.5)
Neonatal feeding difficulties (*n*[%]) (*n* = 50)	42 (84)
Neonatal hypoglycemic (*n*[%]) (*n* = 55)	31 (56.4)
Neurological and developmental features	
Motor delay (*n*[%]) (*n* = 61)	58 (95.1)
Independent walking (*n*[%]) (*n* = 42)	31 (73.8)
Age at which independent walking achieved (months, m[IQR]) (*n* = 27)	19 (17; 30)
Speech delay (*n*[%]) (*n* = 59)	54 (91.5)
Developed speech (*n*[%]) (*n* = 42)	30 (71.4)
First words (months, m[IQR]) (*n* = 20)	24.5 (20; 36)
Intellectual disability (*n*[%]) (*n* = 57)	53 (93)
Intellectual disability severity (*n* = 39)	
Mild (*n*[%])	9 (23.1)
Moderate (*n*[%])	9 (23.1)
Severe (*n*[%])	21 (53.8)
Behavioral problems (*n*[%]) (*n* = 41)	24 (58.5)
Seizures history (*n*[%]) (*n* = 47)	17 (36.2)
Hypotonia (*n*[%]) (*n* = 53)	34 (64.2)
CNS anomalies (*n*[%]) (*n* = 28)	17 (60.7)
Ventriculomegaly (*n*[%])	6 (21.4)
Delayed CNS myelination (*n*[%])	3 (10.7)
Other (*n*[%])	10 (35.7)
Ectodermal anomalies (*n*[%]) (*n* = 48)	45 (93.8)
Fetal fingertip pads (*n*[%])	35 (72.9)
Hypertrichosis (*n*[%])	11 (22.9)
Nail anomalies (*n*[%])	5 (10.4)
Other (*n*[%])	13 (27.1)
Musculoskeletal anomalies (*n*[%]) (*n* = 55)	44 (80)
Brachy/clinodactyly (*n*[%])	24 (43.6)
Joint hypermobility (*n*[%])	23 (41.8)
Hip problems (*n*[%])	14 (25.5)
Other (*n*[%])	15 (27.33)
Gastrointestinal anomalies (*n*[%]) (*n* = 54)	40 (74.1)
Feeding difficulties requiring support (*n*[%])	33 (61.1)
Gastroesophageal reflux (*n*[%])	15 (27.8)
Chronic diarrhea (*n*[%])	3 (5.6)
Recurrent vomiting (*n*[%])	1 (1.9)
Palate anomalies (*n*[%]) (*n* = 67)	43 (64.2)
High/narrow palate (*n*[%])	38 (56.7)
Cleft lip/palate (*n*[%])	8 (11.9)
Velopharyngeal insufficiency (*n*[%])	3 (4.5)
Dental anomalies (*n*[%]) (*n* = 45)	27 (60)
Hypodontia (*n*[%])	10 (22.2)
Malocclusion (*n*[%])	7 (15.6)
Neonatal teeth (*n*[%])	7 (15.6)
Other (*n*[%])	12 (26.7)
Ophthalmological problems (*n* = 48)	28 (58.3)
Strabismus (*n*[%])	15 (31.3)
Nystagmus (*n*[%])	5 (10.4)
Other (*n*[%])	15 (31.3)
Immunological anomalies (*n*[%]) (*n* = 52)	26 (50)
Recurrent infections (*n*[%])	22 (42.3)
Eczema (*n*[%])	4 (7.7)
Other (*n*[%])	4 (7.7)
Cardiovascular anomalies (*n*[%]) (*n* = 63)	31 (49.2)
Septal defects (*n*[%])	15 (23.8)
Valvular anomalies (*n*[%])	11 (17.5)
Aorta anomalies (*n*[%])	10 (15.9)
Other (*n*[%])	13 (20.6)
Endocrine anomalies (*n*[%]) (*n* = 47)	18 (38.3)
Hyperinsulinism (*n*[%])	13 (27.7)
Breast anomalies^d^ (*n*[%])	5 (10.6)
Other (*n*[%])	2 (4.3)
Hearing loss (*n*[%]) (*n* = 26)	8 (30.8)
Genitourinary anomalies (*n*[%]) (*n* = 53)	14 (26.4)
Horseshoe kidneys (*n*[%])	4 (7.5)
Vesicoureteric reflux (*n*[%])	2 (3.8)
Other (*n*[%])	12 (22.6)

The table is arranged according to the highest to lowest frequency of features in individual system.

*CNS * central nervous system, *HC* head circumference, *IQR * interquartile range, *m* median.

^a^The number of responders are detailed for every feature, and their frequencies/distributions were calculated according to that number.

^b^As defined by the Kabuki Syndrome Medical Advisory Board.

^c^At last examination.

^d^It includes gynecomastia and premature thelarche.

**Fig. 2:**
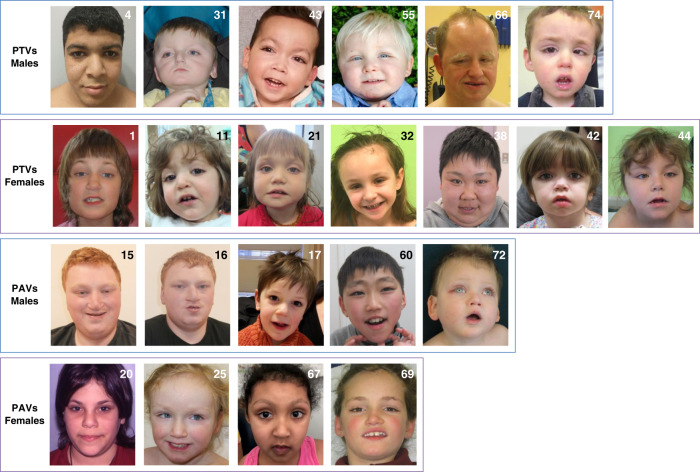
Facial phenotypes of patients with *KDM6A* variants are highly variable. Patients are grouped according to sex and type of variant. Most patients have arched eyebrows, long palpebral fissures, some eversion of the lateral part of the lower eyelids, and bulbous or rounded nasal tip. Unlike the classical gestalt of Kabuki syndrome, the eyebrows are interrupted in only some patients, and are thick in many (P4, 11, 60, 66, and 67) or thin and penciled occasionally (P1, 72). Lateral flaring of eyebrows is observed in some patients (P11, 67, 69, and 74). Ears are not simple or as prominent as usually seen in patients with classic Kabuki syndrome. In several patients they appear relatively large and rather fleshy. Prominent lower lips or pillowing of the lower lips was observed in some patients (P11, 21, 42, 44, 60, 67, and 74). Prominent forehead was observed frequently. Patient 20 inherited the p.Ala149Thr variant form her father, who is similarly affected. PAV  protein-altering variants, PTV protein-truncating variants.

Feeding difficulty was the most frequent neonatal finding. Hypoglycemic was described in 56.4% of neonates (Table 1). The median weight at last examination was −1.43 SD (min = −4, max = 2.28), the median height was −2 SD (min = −7, max = 2.3), and the median head circumference (HC) was −2.34 SD (min = −5.33, max = 2.45). Motor delay was described in 95% of individuals. Overall, 73.8% of patients were reported to have achieved independent walking (Table 1), and 79.4% of patients older than 3 years of age (*n* = 34) had achieved independent walking (Supplementary Table S1). Speech delay was reported in 91.5% of individuals and 71.4% had achieved speech. ID was reported in 93% of individuals with more than half being classified as having severe ID. Congenital malformations affecting the cardiovascular system were most frequent, followed by palatal and renal malformations. Strabismus and hearing loss were the most frequent problem affecting the sensory system. Recurrent infections, hyperinsulinism, seizures, joint hypermobility, and gastroesophageal reflux were some of the other most significant and frequently encountered medical issues. Only 63.7% (*n* = 51) of patients had typical KS facial features, as defined by the diagnostic criteria^2^ (Table 1, Fig. 2).

### Sex differences in KS2

**Fig. 3:**
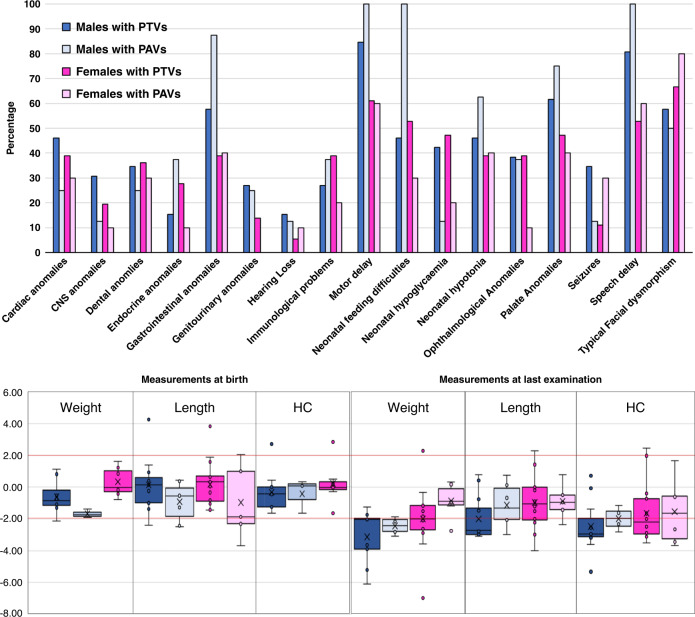
Sex and variant type differences in type 2 Kabuki syndrome. The upper panel shows the comparison of frequency of selected phenotypes between males and females with either PTVs or PAVs. Clinical features are arranged alphabetically from left to right. The lower panel shows the comparison of anthropometric data of patients at birth and at last examination. The *y*-axis denotes SD. Horizontal red lines depict +2 SD and −2 SD. Groups for which we had sparse data (female PAV birth weight and HC) have not been shown in these charts. CNS central nervous system, HC head circumference, PAV protein-altering variants (e.g., missense, in-frame indels), PTV protein-truncating variants (e.g., nonsense, frameshift, splice site).

Next we compared the frequencies of clinical features between male and female patients (Table 2) (Fig. 3a, b). Where full inheritance information was available, de novo variants were found to be significantly more likely in affected females (females = 92.1% vs. males = 62.5%; *p* = 0.007) (Table 2). Affected males were born significantly earlier, and had shorter birth lengths in comparison with female patients (Table 2) (Fig. 3). Males were significantly shorter in stature at the last examination (Table 2) (Fig. 3). Fewer males could walk independently or developed speech (Table 2). Males also significantly more frequently had severe ID (Table 2), (Fig. 3). Males displayed a significantly higher frequency of gastrointestinal problems when compared with females (males = 88% vs. females = 62.1%; *p* = 0.03) (Table 2) (Fig. 3).

**Table 2:** Significant associations between clinical features and sex or variant group.

Feature	Association		*p* value^a^
	Females	Males	
De novo variants (*n*[%])	35 (92.1)	15 (62.5)	0.007
Gestation (weeks, m[IQR])	39 (38; 39)	36 (35.3; 38.5)	0.003
Birth length SD (m[IQR])	−0.01 (−0.41; 1.24)	−1.07 (−1.75; −0.53)	0.013
Height SD (m[IQR])^a^	−1.2 (−2.67; −0.77)	−2.18 (−3.76; −1.94)	0.005
Independent walking (*n*[%])	21 (95.5)	10 (50)	0.001
Developed speech (*n*[%])	20 (90.9)	10 (50)	0.003
ID severity			0.021
Mild (*n*[%])	7 (33.3)	2 (11.1)	
Moderate (*n*[%])	7 (33.3)	2 (11.1)	
Severe (*n*[%])	7 (33.3)	14 (77.8)	
Behavioral problems (*n*[%])	18 (75)	6 (35.3)	0.011
Ectodermal anomalies (*n*[%])	31 (100)	14 (82.4)	0.039
Gastrointestinal problems (*n*[%])	18 (62.1)	22 (88)	0.03
	PAV	PTV	
De novo variants (*n*[%])	8 (57.1)	42 (87.5)	0.02
Age at last examination (years, m[IQR])	12 (6.5; 14.08)	6.35 (1.94; 11.02)	0.043
Birth length SD (m[IQR])	−1.56 (−1.86; −0.9)	−0.29 (−0.98; 0.93)	0.034

*ID * intellectual disability, *IQR * interquartile range, *m*  median, *PAV * protein-altering variant, *PTV * protein-truncating variant.

^a^At last examination.

### Genotype–phenotype correlation in KS2

Next we compared the frequencies of clinical features between patients with PTVs and PAVs (Table 2) (Fig. 3a, b). PTVs were found to be significantly more likely to have occurred de novo (PTVs = 87.5% vs. PAVs= 57.1%; *p* = 0.02) (Table 2). Age of last medical examination of individuals with PTVs was significantly earlier in comparison with individuals with PAVs (Table 2). Individuals with PAVs had shorter birth lengths in comparison with individuals with PTVs (Table 2) (Fig. 3a, b). There was no association between sex of the affected individuals and the type of variants (PAVs in males = 23.5%; PAVs in females = 21.7%; PTVs in males = 76.5%; PTVs in females = 78.3%; *p* = 0.85).

## DISCUSSION

To date, this is the largest study of individuals with pathogenic *KDM6A* variants, which allows delineation of the variant spectrum, the clinical features of KS2, and allows us to determine the sex differences and genotype–phenotype correlations. The age range of our cohort and the sex distribution suggests that these data are likely to be representative of most patients seen in clinics.

### Spectrum of pathogenic *KDM6A* variants

This study substantially expands the known spectrum of pathogenic *KDM6A* variants; 87.5% (*n* = 70) of the individuals in this study have *KDM6A* point variants (Fig. 1, Supplementary Figure 2A, Supplementary Table S1), which is in contrast with the initial description of large deletions.^6^ However, this is reflective of our recruitment criteria that resulted in exclusion of large CNVs.

We found that 72.9% (*n* = 62) individuals had *KDM6A* PTVs (Fig. 1 and Supplementary Figure 2A) (Supplementary Table S1), which is similar to our observations in several other disorders caused by variants in histone lysine methylases and demethylases.^1^ We found the *KDM6A* PTVs to be distributed throughout the gene, from exon 1 to exon 27 in both male and female cohorts.

Previously, only five distinct pathogenic *KDM6A* PAVs have been published and our study substantially increases this number. Pathogenic PAVs were mainly found to cluster in the TRP 2, TRP 3, TRP 7 and Jmj-C domains of KDM6A (Fig. 1). However, the p.(Ser1025Gly) and the p.(Lys1080_1083delinsGlySer) variants are located outside any known domains.^21^ The p.(Arg1255Trp) variant, located in the JmjC domain, was seen in two unrelated individuals, and was proven to be de novo in one patient.^17^ The variants c.445G>A (p.[Ala149Thr]), c.563A>G (p.[Lys188Arg]) and c.974G>C (p.[Gly325Ala]) affect the nucleotides near the exon–intron boundaries and could potentially affect normal splicing. We could not test effect of these variants on splicing as part of this study. These results should facilitate interpretation of *KDM6A* variants in clinics. In the future, functional analysis using DNA methylation signatures^22–25^ or epigenetic reporter assays^26^ might be useful to determine the significance of some PAVs. In future, systematic comparison of *KDM6A* germline and somatic missense variants, as recently performed for *KMT2D*, might also be possible.^27^

### Inheritance of pathogenic *KDM6A* variants

Although a vast majority of pathogenic *KDM6A* variants occurred de novo, we found 12 cases from 9 families with inherited pathogenic variants. Seven (4 PTVs and 3 PAVs) were inherited from similarly affected mothers. In other cases, the phenotype information of the mother was not available. Notably, where complete inheritance information was available, inherited variants constituted variants of 59% males of our cohort and 42.9% of PAVs. These figures might be overestimated due to inheritance information not being available in 25% of the cohort (assuming that the parents who appeared unaffected are less likely to be tested). However, our data clearly show that some women with KS2 can have children. One affected boy inherited a mosaic LoF *KDM6A* variant from his unaffected mother. Interestingly, we also detected one pathogenic PAV p.(Ala149Thr) inherited from a similarly affected father. Paternally inherited pathogenic *KDM6A* variant has never been described previously. Together these findings emphasize the importance of parental testing in patients with pathogenic *KDM6A* variants and have important implications in clinical practice and counseling.

### Antenatal and neonatal phenotype of pathogenic *KDM6A* variants

Our data suggest that intrauterine growth retardation (IUGR) is the most frequent significant prenatal finding in patients with pathogenic *KDM6A* variants (Table 1 and Supplementary Table S1). IUGR was present in 6.25% of patients in our total cohort and in 18.5% patients where prenatal information was available. Fewer than 10% of patients with KS1 and/or clinically diagnosed KS have IUGR.^28^ Interestingly 11 of 13 patients in our cohort known to have been born prematurely (before 37 weeks of gestation) were males. Birth length and HC in patients with pathogenic *KDM6A* variants were observed to be in normal-to-low range. Males with pathogenic *KDM6A* variants appear to have significantly smaller birth lengths.

### Growth and development in patients with pathogenic *KDM6A* variants

Among patients with clinically diagnosed KS, 55–71% have short stature and 25–32% have microcephaly.^29–31^ In our cohort of patients with pathogenic *KDM6A* variants short stature was less frequent (48%) and microcephaly was more frequent (54%) (Supplementary Table S1). Comparisons of SDs of weights, lengths/heights, and HCs at last examination against measurements at birth, clearly reveal that the growth retardation in this condition is mostly of postnatal origin. The distribution of height SDs reveals a trend across the four groups (males with PTVs < males with PAVs < females with PTVs < females with PAVs). Hence, the sex and the type of variant should be considered in growth-related prognosis and treatment of patients with KS2. Similar to what is seen in KS1, 5/9 patients in our cohort with age of >15 years had a body mass index >25 kg/m^2^ (Table 1) suggesting a tendency to be overweight or obese with age,^32,33^ that may have important medical implications. Larger data sets from adults with KS2 is required to enable correlation with sex and variant types.

Developmental delay and/or intellectual disability is reported in over 84%^30^ patients with clinically diagnosed cases of KS. In our cohort these phenotypes were found in 95% of patients (Table 1 and Supplementary Table S1). Differences in the ascertainment criteria of historical studies of KS make comparisons with our data challenging. However, it is clear that the frequency and the severity of neurodevelopmental problems in males with pathogenic *KDM6A* variants are significantly greater. Males have significantly lower levels of independent walking and speech. Of note, there was no significant difference in the ages at last examination of male and female patients in this cohort (*p* = 0.924). Notably, the developmental phenotype of females was much more variable than in males. This variability in presentation could be due to differences in X-chromosome inactivation in females. However, systematic X-chromosome inactivation studies will be needed to confirm this. This is particularly interesting because *KDM6A* is known to partially escape X-inactivation.^6^

Patients with PTVs tended to have more intellectual disability (97.6% versus 80%, *p* = 0.052) and higher frequency of central nervous system (CNS) anomalies (71.4% versus 28.6%, *p* = 0.076), although the difference did not reach statistical significance. Overall, individuals with PTVs have a more severe phenotype, and the phenotypes of patients with PAVs was more variable. Of note, the proportion of PTVs and PAVs was not significantly different between male and female patients. Also, the phenotype variability seen in patients with PAVs could perhaps be explained by allelic heterogeneity, differences in the genetic background, or other multifactorial effects. Although most pathogenic *KDM6A* PAVs were located in known functional domains, the demethylase-independent mechanism for some PAVs^20^ might also explain these differences. It must be noted that we have not collected scores of formal developmental and neuropsychological assessments. In future, systematic studies could provide more objective assessments in these domains.

### Congenital and sensory anomalies in patients with pathogenic *KDM6A* variants

Cardiovascular anomalies were reported in 49.2% of patients of our cohort who underwent echocardiogram (Table 1 and Supplementary Table S1). This appears to be higher than the reported frequency of 37–42% in cases of KS.^29,30^ The commonest congenital heart defect was atrial septal defect, followed by ventricular septal defect. Aortic anomalies such as coarctation, bicuspid valve, and stenosis were also frequent. One individual, who was 11 years old, was also reported to have aortic dilatation.

The prevalence of genitourinary anomalies (e.g., kidney and urinary tract malformations, hypercalciuria, abnormal genitalia) was also higher in our cohort (26.4%) (Table 1 and Supplementary Table S1) than what has been previously reported in KS. However, the frequency of kidney/renal tract malformations was lower in our study (only in 11.3% of patients) when compared with previous reports (20–38%).^14,30,31^ The most frequent renal malformation observed in our cohort is horseshoe kidneys.

The frequencies of palate and dental anomalies were high in our cohort (64.2% and 60%, respectively) (Table 1 and Supplementary Table S1), but the presence of cleft lip/palate and hypodontia was lower (11.9% and 22.2%, respectively) when compared with previous reports (35–50% and 48–85%, respectively).^29–31,34^

Around one third (31.3%) of patients in our cohort have strabismus, which has been reported in 21–36% of patients of with KS.^29,30,35^ Interestingly, around 11% of patients in our cohort reported nystagmus. The basis of nystagmus in KS2 patients is unclear and needs further investigation. One individual was reported to have microphthalmia and chorioretinal coloboma, which has also been previously described for *KMT2D*.^36^

Hearing loss affected 30.8% of individuals of our cohort, similar to the 27–43% of reported patients with KS.^30,31^ Information on type of deafness was not available for many patients and, therefore, we did not perform any subgroup analysis.

The frequencies of anomalies depicted here must be interpreted with caution because of the differences in the levels of available clinical details from different centers. To calculate the frequencies of individual features, we removed the patients for whom unequivocal data for presence or absence of that particular feature were unavailable (e.g., patients who had not undergone echocardiogram were not used to calculate the frequency of congenital heart defects in the cohort). This may erroneously inflate the frequency of some clinical features in our cohort (presuming that when not investigated, absence of feature is more likely than its presence). However, broadly these figures are still likely to be useful indicators and will facilitate appropriate management and surveillance of patients with pathogenic *KDM6A* variants. These observations also emphasize the important role of *KDM6A* in embryonic development.

### Other systemic problems in patients with pathogenic *KDM6A* variants

Endocrine abnormalities were seen in 38.3% of patients with pathogenic *KDM6A*. Specifically, we detected a lower frequency of premature thelarche (6.4%) when compared with previous studies (25–43%).^30,31,37^ A higher prevalence of neonatal hypoglycemic (56.4% of the overall cohort) and hyperinsulinism (27.7%) were detected in our cohort. Only 7% of KS cases show transient/persistent hypoglycemia.^30,37^ The higher prevalence of hypoglycemia and hyperinsulinism in KS2 has been suspected previously.^6,8,17^ Notably, inhibition of KDM6A increases the release of insulin from mouse pancreatic islets.^38^

Recurrent infections were reported in 42.3% of patients in our cohort, similar to 48–69% of patients with KS of previous studies.^29,31,37^ We did not collect data about specific immune profiles, types and severity of infections, or responses to treatments. These need to be studied in more detail in the future. Of note, one patient was reported to have vitiligo and one to have hypothyroidism. Notably, various autoimmune features have been reported in patients with KS1.^13^ Gastrointestinal problems, especially feeding difficulties (requiring use of nasogastric tube or gastrostomy), were reported in 61.1% of patients of our cohort. Gastrointestinal problems have been described in 29–74% of patients with clinically diagnosed KS.^31,37^ Males have higher frequency of gastrointestinal problems.

Brachy/clinodactyly and joint hypermobility were the most prevalent musculoskeletal anomalies, which were described in 80% of patients of our cohort, which is similar to ~88% of reported patients with KS.^29–31^ No cases of multiple joint dislocations were recorded in our study. Ectodermal abnormalities such as persistent fingertip pads were detected in 72.9% of patients in our work, which have been described in 82–92% of reported patients with KS.^29–31,39^

### Facial dysmorphism in patients with pathogenic *KDM6A* variants

Facial dysmorphism is considered to be the most distinguishing feature of KS1.^40^ However, only 63.7% of patients in our cohort had typical facial features of KS (Fig. 2). There was no obvious association or pattern of presence of typical facial features and the sex of the patient or the type of variant. However, some individuals with PAVs in TPR regions may have less typical facial dysmorphism (see individuals 15, 16, 17, and 20 in Fig. 2). These observations suggest that atypical facial dysmorphism, on its own, may be insufficient to rule out pathogenicity of a VUS in *KDM6A*.

### Conclusion

This study substantially extends the spectrum of pathogenic *KDM6A* variants and delineates the clinical phenotype of KS2. We demonstrate that males and patients with PTVs tend to be more severely affected. We show the overlaps and differences between the phenotypes of KS2 and KS1. These findings will impact on diagnosis, counseling, monitoring, and treatment of patients with KS2. They also highlight areas of future clinical research need in KS2 and will lead to evidence-based clinical management guidelines for patients.

## Supplementary information

Supplementary FigureS1

Supplementary TableS1

Supplementary TableS2

## Data Availability

All clinical and genetic data included in this study are provided in Supplementary Table S1.
